# Panniculitis Is an Important Feature of Cutaneous Leishmaniasis Pathology

**DOI:** 10.1155/2012/612434

**Published:** 2012-04-10

**Authors:** Khalifa E. Sharquie, Ammar Faisal Hameed

**Affiliations:** ^1^Scientific Council of Dermatology & Venereology, Iraqi Board for Medical Specializations, Baghdad 12114, Iraq; ^2^Iraqi Society of Dermatology & Venereology, Medical Collection Post Office, P.O. Box 61080, Baghdad 12114, Iraq; ^3^Department of Dermatology, College of Medicine, Baghdad University, Baghdad, Iraq

## Abstract

*Background*. Cutaneous leishmaniasis is an inflammatory parasitic infection characterized by superficial and deep perivascular infiltration with or without granuloma formation. Clinical diagnosis usually requires seeing *Leishmania* bodies. *Methods*. We report two cases of cutaneous leishmaniasis with unusual histological finding of panniculitis. Case 1: a 36-year-old male presented with multiple ulcerative nodules involving the left leg for two months duration which was greatly responsive to antimony intralesional therapy. Case 2: A 45-year-old woman presented with painless nodules on her upper chest of a 10-week duration which were successfully treated with oral and topical zinc sulphate. *Results*. Diagnosis of both cases was confirmed by finding the Leishmania bodies with Gimesa stain in addition to the diffuse dermal inflammatory cellular infiltration of the dermis forming granulomatous dermatitis. Mixed cellular infiltration of lymphocytes, histiocytes, and plasma cells of the panniculus caused both septal and lobular panniculitis. *Conclusion*. Cutaneous leishmaniasis can cause panniculitis and this could be seen more commonly if deep biopsies were taken. So cutaneous leishmaniasis must be considered in evaluating pathology of panniculitis especially in endemic regions.

## 1. Introduction

Cutaneous leishmaniasis (CL) is a disease caused by intracellular protozoa *Leishmania* parasite. Infection is transmitted by the bite of a sand fly. In Iraq, the clinical spectrum of CL ranges from a self-resolving cutaneous ulcer to a dry granulomatous lesion and very rarely a disseminated cutaneous disease [1]. The definitive diagnosis depends on demonstration of the parasites by smears, culture, PCR, and histopathological investigation [2, 3]. The histological spectrum of CL ranges between diffuse dermal mixed inflammatory cell infiltrate to granuloma formation [3–5]. There are only two reports in the medical literature that demonstrated panniculitis in lesions of CL [6, 7].

The aim of the present study is to report two cases of CL associated with panniculitis by histopathological examination.

### 1.1. Case 1

 A 36-year-old male patient presented to our department with painless nodules on his left foot for a two-month duration for which he received a three-week course of systemic (ampicillin-cloxacillin) antibiotic without any improvement. His medical and family history were unremarkable.

**Figure 1:**
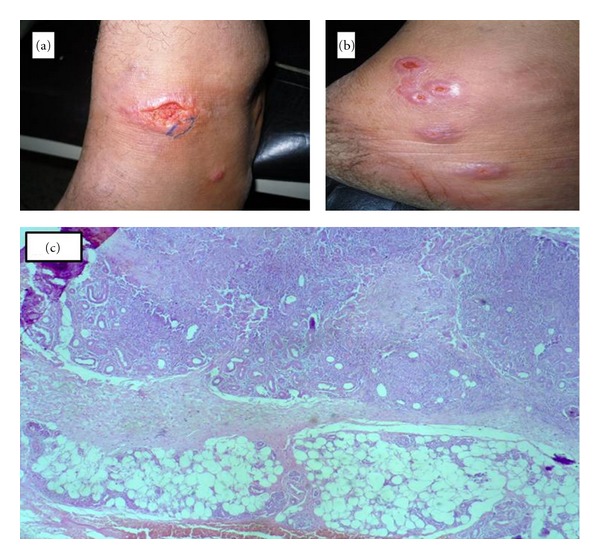
Ulcerative plaque on the dorsal foot, the blue elliptical line indicates the site of the biopsy (a), multiple ulcerative nodules behind the lateral malleolus (b), microscopic view of septal and lobular panniculitis H&E stain ×100 (c).

Dermatological examination revealed a large ulcerative plaque on the dorsal aspect of the left foot surrounded by two satellite purple papules (Figure 1(a)). In addition to multiple grouped dusky erythematous nontender ulcerative nodules behind the lateral malleolus of the left leg (Figure 1(b)). Histopathological examination of the margin of the ulcerative plaque showed ulceration of the epidermis, diffuse dense mononuclear cellular infiltration of lymphocytes, histiocytes, and plenty of plasma cells throughout the dermis with early granuloma formation. These changes even involved the panniculus causing septal and lobular panniculitis (Figure 1(c)).

 The *Leishmania* tropica (LT) bodies were seen in numerous numbers inside the macrophages of superficial dermis in sections stained with H&E and Gimesa. Some of them were extracellular. These bodies were distributed in the dermis in a form of foci. They were absent in the lesions of panniculitis.

The patient was given 1 mL intralesional sodium stibogluconate (100 mg per 1 mL) for each lesion weekly for 5 successive weeks with excellent gradual resolution of all skin lesions [8].

### 1.2. Case 2

A 45-year-old female patient presented with painless nodules on the anterior upper chest for a 10-week duration. She was treated with oral (amoxicillin-clavulanic acid) antibiotic for two weeks without any response, otherwise she was completely healthy.

On examination, she had two nontender, nonulcerative, indurated dusky erythematous plaques on her upper anterior chest. Complete workup included chest XR, complete blood film, liver function test, renal function test, and abdominal sonography, all these investigations were normal.

**Figure 2:**
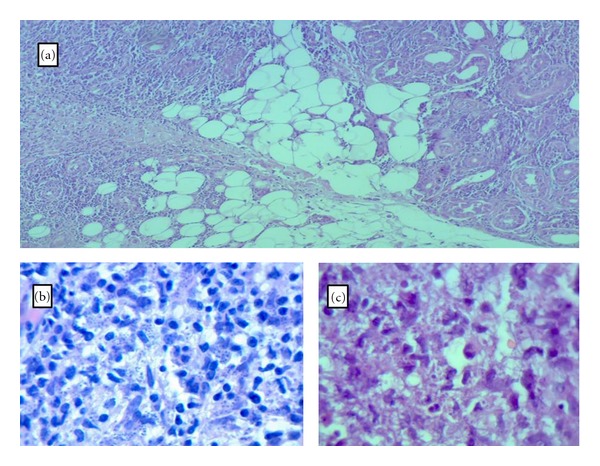
Microscopic view of septal and lobular panniculitis H&E ×400 (a), dermal foci of intra and extracellular LT bodies H&E ×1000 (b), non-metachromatic LT bodies Gimesa stain ×1000 (c).

A 6 mm punch biopsy was taken from one of the nodules which showed acanthotic epidermis and diffuse dermal infiltration of mononuclear lymphocytes and histiocytes, while plasma cells were present in high numbers. There was a tendency for granuloma formation. The fatty lobules and its septa were infiltrated by lymphocytes, histiocytes, and plasma cells causing both septal and lobular panniculitis (Figure 2(a)).

Aggregations of high number of LT bodies were seen mainly inside of macrophages of the superficial dermis stained by H&E (Figure 2(b)). Gimesa stain confirmed the presence of the non-metachromatic nuclei of the LT bodies (Figure 2(c)). These bodies were noticed to be distributed in foci fashion. LT bodies were not seen in the area of panniculitis.

The patient showed good clinical response to topical zinc sulphate solution 20% twice daily application combined with oral zinc sulphate 200 mg thrice daily for a one-month duration [9].

## 2. Discussion

Cutaneous leishmaniasis is a major public health problem in many countries including Iraq [8]. Two species of *leishmania* have been isolated in Iraq:* Leishmania tropica* and *leishmania major*. Both are implicated in the development of wet and dry lesions characterized by early ulceration and late ulceration, respectively [10]. The histopathology of CL has been described as a spectrum, at one end, especially in the early ulcerative lesions there is superficial and deep perivascular lymphocytic infiltration with plenty of plasma cells together with foci of LT bodies. At the other end of the spectrum especially in the late dry lesion, there is granuloma formation with few plasma cells, and no LT bodies can be detected [3]. Panniculitis is not reported as part of histopathology of CL. However, it is well documented that chronic infection is an important cause of panniculitis, for example *mycobacterial* panniculitis [11].

 Recently two reports appeared in the literature, They described the histological finding of panniculitis in CL [6, 7]. Meziou et al. showed disseminated lesions of CL secondary to lymphedema with superimposed erysipelas. In their two diabetic patients, the histological examination of skin biopsy samples revealed lobular panniculitis [6].

Eryilmaz et al. demonstrated a case of leishmaniasis with histopathology of lobular panniculitis of granulomatous pattern [7].

The present work described two cases with characteristic panniculitis which is both septal and lobular in lesions of CL. LT bodies were not detected in the panniculitis lesion whereas the infective pathogen was usually engulfed by the dermal macrophages. So panniculitis can be considered as part of the inflammatory reaction in CL especially in the early ulcerative lesions. We think that panniculitis is not rare finding in CL, but it is easily missed because most biopsies are superficial rather than deep where the subcutaneous tissue is not included in the examination material. Accordingly further study will be arranged to include all CL lesions with deep biopsies involving the fatty layer of the skin.

In conclusion, panniculitis must be considered as an important histopathological feature of lesions of CL, and deep biopsies are always suggested so that it cannot miss this important inflammatory reaction of the disease.
